# Supplementary material for: Salmonella spp. in Aquaculture: An Exploratory Analysis (Integrative Review) of Microbiological Diagnoses between 2000 and 2020
Source: Animals (Basel). 2022 Dec 21;13(1):27. doi: 10.3390/ani13010027 (PMC9817981; doi:10.3390/ani13010027)
Supplement: Supplementary file 1 [file animals-13-00027-s001.zip › animals-2085568-supplementary.pdf]

Table S1. Characteristics of the articles selected in the integrative review on the microbiological diagnoses of *Salmonella* spp. in aquaculture between 2000 and 2020.

Authors, year[reference]	Country	Sample			Tested	Diagnosis assay	*Salmonella*	
		Source(s)	Specie(s)/ Environmental(s)	Type	Total	Method(s)	n° positive (%)	Sorotype(s)
Esposto, E. M. et al. (2007)[23]	Brazil	Aquaculture	Fish (*Oreochromis niloticus*)	Body<br>Tissue<br>Viscera	45	Culture**	4(8.9%)	O:17<br>*S.* spp.
Pilarski, F. et al. (2004)[9]	Brazil	Aquaculture	Fish (*Cyprinus carpio*)<br>Fresh water	Tissue<br>Environment	264	Culture**	Nd	
Araujo, W. S. C. et al. (2017)[72]	Brazil	Aquaculture, industry	Fish (*Colossoma macropomum*)	Tissue	112	Culture**	Nd	
Calixto, F. A. A. et al. (2016)[73]	Brazil	Aquaculture, market	Fish (*Rachycentron canadum*)	Tissue	11	Culture*	Nd	
dos Santos, R. R. et al. (2019)[18]	Brazil	Aquaculture	Fish (*Colossoma macropomum*, *Leiarius marmoratus* × *Pseudoplatystoma reticulatum*, *Brycon orbignyanus*, *Piaractus mesopotamicus*, *Leporinus obtusidens*)<br>Fresh water<br>Feces	Body<br>Feces<br>Environment	567	Culture**<br>PCR	39(6.87%)	Enterica<br>Arizonae<br>Brandemburgo<br>Hadar<br>Heidelberg<br>Panama<br>Saintpaul

Palhares, J. C. P. et al. (2014)[74]	Brazil	Aquaculture	Fresh water	Environment	48	Culture*	35(73%)	Panama<br>Infantis<br>Typhimurium
Pastro, D. C. et al. (2019)[75]	Brazil	Aquaculture, market	Fish (*Colossoma macropomum* x *Piaractus brachypomus*, *Colossoma. macropomum* x *Piaractus mesopotamicus*, *Pimelodus maculatus*)	Tissue	56	Culture*<br>PCR	4(7.14%)	*S.* spp.
Ribeiro, R. V. et al. (2010)[76]	Brazil	Aquaculture	Fish (*Oreochromis niloticus*)<br>Fresh water	Tissue<br>Environment	111	Culture*	9	Mbandaka<br>Agona<br>Anatum<br>Havana<br>Livingstone<br>Others (n = 15)
Klase, G. et al. (2019)[25]	China	Aquaculture	Fresh water	Environment	27	Culture**<br>qPCR	10(37%)	*S.* spp.
Broughton, E. I. et al. (2009)[42]	China	Aquaculture, market	Fish (species not described)	Tissue	100	Culture*	5(5%)	*S.* spp.
Li, K. et al. (2017)[17]	China	Aquaculture	Fish (*Oreochromis niloticus*)<br>Fresh water	Body<br>Viscera<br>Environment	59	Culture*<br>PCR	16(27%)	Weltevreden<br>4,5,12:i:- monophasic variant of S. Typhimurium<br>Stanley
Li, Y. et al. (2019)[43]	China	Aquaculture, market	Fish (species not described),	Body<br>Tissue<br>Viscera	3226	Culture**	124(3.8%)	Stanley<br>Thompson<br>Lichfield

			Crustaceans (species not described), Mollusks (species not described)					Typhimurium<br>Virchow<br>Carrau<br>Infantis<br>Others (n = 10)
Pawar, P. P. et al. (2020)[44]	China	Industry	Fish (*Catla catla*)	Tissue	NA	Culture*	Nd	
Yang, X. et al. (2015)[45]	China	Aquaculture, market	Fish (species not described), Shrimp (species not described), Oyster (species not described)	Tissue<br>Viscera	554	Culture**	86(15.5%)	Typhimurium<br>Wandsworth<br>Thompson<br>Derby<br>Pomona<br>Lichfield<br>Weltevreden<br>Enteritidis<br>Others (n = 32)
Zhang, J. et al. (2015)[46]	China	Aquaculture	Fish (species not described), Shrimp (species not described), Bivalve mollusks (species not described), Shellfish (species not described)	Tissue<br>Viscera	730	Culture*	217(29.7%)	Alberdeen<br>Wandworth<br>Thompson<br>Cingapura<br>Stanley<br>Schwarzengrund<br>Hvittingfoss<br>Typhimurium<br>Others (n = 30)
Surendraraj, A. et al. (2009)[47]	India	Aquaculture	Fish (*Catla catla*, *Labeo rohita*, *Cirrhinus cirrhosus*, *Ctenopharyngodon Idella*)<br>Fresh water	Tissue<br>Environment	88	Culture*	4(3.1%)	*S.* spp.

			Salt water<br>Sediment					
Kumar, R. et al. (2009)[48]	India	Market	Fish (species not described),<br>Shrimp (species not described),<br>Mussel (species not described),<br>Crab (species not described),<br>Oyster (species not described),<br>Squid (species not described),<br>Cuttlefish (species not described),<br>Octopus (species not described),<br>Clam (species not described)	Tissue	247	Culture*	69	Weltevreden<br>Typhimurium<br>Braenderup<br>Newport<br>Others (n = 9)
Kakatkar, A. S. et al. (2011)[49]	India	Market	Fish (*Schilbe mystus*,<br>*Clarias gariepinus*,<br>*Tenualosa*,<br>*Catla catla*,<br>*Labeo rohita*,<br>*Harpadon nehereus*,<br>*Engraulidae*),<br>Prawns (species not described)	Tissue	NA	Culture*	65	Typhimurium<br>Oslo<br>Ohio<br>Weltevreden<br>Tennessee
Patel, A. et al. (2020)[50]	India	Aquaculture	Fresh water<br>Sediment	Environment	188	Culture*<br>PCR	12	*S.* spp.

Saharan, V. V. et al. (2020)[51]	India	Aquaculture	Fish (species not described)	Feces	160	Culture* PCR	72(45%)	*S.* spp.
Shabarinath, S. et al. (2007)[52]	India	Market	Fish (species not described), Shrimp (species not described), Oyster (species not described), Clam (species not described), Salt water	Body Environment	100	Culture* PCR	52(52%)	Weltevreden Worthington Newport
Shakila, R. J. et al. (2012)[53]	India	Aquaculture, industry	Fish (*Rachycentron canadum*)	Tissue	2	Culture**	Nd	
Martinez, O. et al. (2009)[27]	Spain	Aquaculture, market	Bivalve (*Mytilus galloprovincialis, Venerupis pullastra, Ruditapes philippinarum, Dosinia exoleta, Cerastoderma*)	Tissue	53 batches	Culture** PCR	Nd	
Álvarez, A. et al. (2008)[62]	Spain	Aquaculture, market	Fish (*Sparus aurata*)	Tissue	NA	Culture**	Nd	
Doménech, E. et al. (2015)[63]	Spain	Industry	Fish (species not described)	Tissue	205	Culture*	Nd	
Hernández, M. D. et al. (2009)[64]	Spain	Aquaculture	Fish (*Argyrosomus regius*)	Tissue	NA	Culture**	Nd	
Martinez-Urtaza, J. et al. (2005)[65]	Spain	Industry	Bivalve (species not described)	Tissue	NA	Culture**	106	Senftenberg
Peres Costa, J. C. C. (2020)[66]	Spain	Aquaculture	Fish (*Sparus aurata,*	Body Viscera	95	Culture* PCR	Nd	

			Dicentrarchus labrax)					
Zhao, S. et al. (2003)[14]	USA	Imported food	Seafood (species not described)	NA	4072	Culture** PCR	187	Weltevreden Thompson Lexington Newport Enteritidis Arizonae Typhimurium Others (n = 74)
Akiyama, T. et al. (2011)[77]	USA	Imported food	Seafood (species not described)	Tissue	NA	Culture*	39	Saintpaul
Pal, A. et al. (2009)[78]	USA	Aquaculture	Fish (*Ictalurus punctatus*, *Pangasius bocourti*)	Tissue	60	Culture**	25(42%)	*S.* spp.
Ponce, E. et al. (2008)[79]	USA	Imported food	Fish (species not described), Shrimp (species not described), Crabs (species not described), Snails (species not described), Mussel (species not described)	Tissue	NA	Culture*	210	Weltevreden Newport Saintpaul Senftenberg Lexington Virchow Bareily Others (n = 57)
Wang, F. et al. (2011)[80]	USA	Imported food	Fish (species not described), Shrimp (species not described)	Tissue	171	Culture** PCR	30(17.5%)	Typhimurium
Mahmoud, M. et al. (2016)[85]	Egypt	Aquaculture	Fish (*Oreochromis niloticus*, *Mugil cephalus*)	Body Viscera Environment		Culture*	yes	*S.* spp.

			Fresh water					
Elsaidy, N et al. (2015)[84]	Egypt	Aquaculture	Fish (*Oreochromis niloticus*)<br>Fresh water	Tissue<br>Environment	29	Culture*	11	*S.* spp
Abou-Elela, G. et al. (2009)[83]	Egypt	Aquaculture	Salt water<br>Sediments	Environment		Culture**	3	Pullorum<br>Choleraesuis
Rubini, S. et al. (2018)[68]	Italy	Market	Bivalve (*Ruditapes philippinarum, Mytilus galloprovincialis, Chamelea gallina, Crassostrea gigas, Ostrea edulis*)<br>Salt water	Tissue<br>Environment	10757	Culture*	237(2,2%)	Typhimurium<br>Monophasic variant 4,[5],12:i:-<br>Derby<br>Newport<br>Thompson<br>Others (n = 48)
Smaldone, G. et al. (2017)[69]	Italy	Aquaculture, industry	Fish (*Trachurus trachurus, Oncorhynchus mykiss*)	Tissue	NA	Culture*	Nd	
Caruso, G. et al. (2004)[67]	Italy	Aquaculture	Fish (*Dicentrarchus labrax L., Sparus aurata L*)<br>Salt water<br>Sediment	Tissue<br>Environment	193	Culture*	Nd	
Sing, C. et al. (2016)[56]	Malaysia	Aquaculture, market	Fish (*Clarias gariepinus*)	Body<br>Viscera	60	Culture**<br>PCR	6(10%)	Corvalis<br>Mbandaka<br>Typhimurium
Budiati, T. et al. (2013)[55]	Malaysia	Aquaculture, market	Fish (*Clarias gariepinus, Tilapia mossambica*)<br>Fresh water	Tissue<br>Viscera<br>Environment	172	Culture**	34	Albany<br>Corvalis<br>Agona<br>Stanley<br>Typhimurium<br>Mikawashima

								Bovismobificans<br>*S.* spp.
Banerjee, S. et al. (2012)[54]	Malaysia	Aquaculture	Shrimp (*Litopenaeus vannamei*)<br>Fresh water	Tissue<br>Viscera<br>Environment	183	Culture**	4	Corvalis
Yen, N. et al. (2020)[59]	Vietnan	Market	Shrimp (*Litopenaeus vannamei*, *Penaeus monodon*, *Penaeus merguiensis*, *Metapenaeus ensis*, *Macrobrachium rosenbergii*)	Body	40 lotes	Culture*	30 lotes (75%)	Braenderup<br>Anatum<br>Saintpaul<br>Rissen<br>Litchfield
Nguyen, D. et al. (2016)[57]	Vietnan	Aquaculture, market	Fish (species not described), Shrimp (species not described)	Tissue<br>Viscera	154	Culture**<br>PCR	63	Rissen<br>Weltevreden<br>Londres<br>Anatum<br>Typhimurium<br>Corvalis<br>Others (n = 46)
Uddin, G. et al. (2015)[58]	Vietnan	Aquaculture	Shrimp (*Littopenaeus vannamei*)	Tissue<br>Viscera	48	Culture*<br>PCR	11	Weltevreden<br>Agona
Aubourg, S. et al. (2007)[81]	Chile	Aquaculture	Fish (*Oncorhynchus kisutch*)	Tissue	40	Culture**	Nd	
Dondero, M. et al. (2004)[82]	Chile	Industry	Fish (*Salmo salar*)	Tissue		Culture*	Nd	
Brenner, M. et al. (2009)[71]	Germany	Aquaculture	Bivalve (*Mytilus edulis* L.)	Tissue	5	Culture*	Nd	

Atanassova, V. et al. (2008)[70]	Germany	Market	Fish (*Salmo salar*)	Tissue	250	Culture*	3(2,4%)	*S.* spp.
Wanja, D. et al. (2020)[89]	Kenya	Aquaculture	Fish (*Oreochromis niloticus*, *Clarias gariepinus*, *Carassius auratus*, *Cyprinus carpio carpio*)<br>Fresh water	Viscera<br>Environment		Culture**	4	Enteritidis
Miruka, D. et al. (2013)[90]	Kenya	Aquaculture	Fish (*Oreochromis niloticus*)<br>Fresh water	Viscera<br>Environment	93	Culture**	5	*S.* spp.
Efuntoye, M. et al. (2012)[86]	Nigeria	Aquaculture	Fish (*Clarias gariepinus*)<br>Fresh water	Body<br>Viscera<br>Environment	108	Culture**	18	Typhimurium<br>Enteritidis<br>*S.* spp.
Akinjogunla, O. et al. (2011)[12]	Nigeria	Market	Fish (*Ethmalosa fimbriata*)	Tissue<br>Viscera	60	Culture**	40	*S.* spp.
Dhowlaghar, N. et al. (2018)[60]	Thailand	Imported food	Fish (species not described)	Tissue		Culture**	yes	Hadar<br>Virchow<br>Blockley
Upadhyay, B. et al. (2010)[61]	Thailand	Aquaculture, market	Shrimp (*Paeneus monodon, Paeneus vannamei*)	Tissue	100	Culture*<br>PCR	24	Typhimurium<br>Anatum<br>Weltevreden<br>Virchow<br>Others (n = 15)
Abbassi-Ghozzi, I. et al. (2012)[87]	Tunisia	Market	Fish (species not described)	Tissue	12	Culture**<br>PCR	Nd	
Boulares, M. et al. (2011)[88]	Tunisia	Aquaculture	Fish (*Chelon labrosus*,	Tissue<br>Viscera	80	Culture**<br>PCR	Nd	

			Merlangius merlangus, *Solea solea*, *Sardina pilchardus*, *Scomber scombus*, *Mullus surmuletus*, *Sparus pagrus*)					
Yildirim, Z. et al. (2018)[92]	Turkey	Aquaculture	Fresh water	Environment		Culture**	yes	Typhimurium Enteritidis
Bingol, E. et al. (2008)[91]	Turkey	Market	Bivalve (*Midye Dolma*)	Tissue	168	Culture**	Nd	
Dib, A. et al. (2018)[21]	Algeria	Market	Fish (species not described), Shrimp (species not described)	Tissue	150	Culture*	2	Infantis
Mannan, M. et al. (2020)[93]	Bangladesh	Aquaculture	Fish (*Oreochromis niloticus*)	Viscera	380	Culture**	52(13,68%)	*S.* spp.
Huys, G. et al. (2007)[94]	Belgium	Aquaculture	Fish (*Aristichthys nobilis*, *Oreochomis niloticus*, *Oreochomis mossambicus*, *Lates calcarifer*, *Clarias macrocephalus*, *Clarias gariepinus*, *Pangasius hypophthalmus*, *Cyprinus carpio*, *Trichogaster pectoralis*,	Viscera Environment	204	Culture** PCR	3	Arizonae *S.* spp.

			Osphronemuss goramy), Shrimp (*Penaeus monodon*), Fresh water Sediment					
Kaktcham, P. et al. (2017)[95]	Cameroon	Aquaculture	Fish (*Oreochromis niloticus*, *Cyprinus carpio*) Fresh water	Viscera Environment	400	Culture* PCR	yes	*S.* spp.
Ayazo-Genes, J. et al. (2019)[96]	Colombia	Aquaculture	Fresh water Sediment Biofloc	Environment Biofloc		Culture**	yes	*S.* spp.
Krog, J. et al. (2014)[97]	Denmark	Aquaculture, market	Bivalve (*Mytilus edulis*)	Tissue	29	Culture**	Nd	
Sagoo, S. et al. (2007)[98]	England	Market	Crustaceans (species not described), Molluskan (species not described), Shellfish (species not described)	Body	682 batches	Culture**	Nd	
Ampofo, J. et al. (2003)[28]	Ghana	Aquaculture	Fish (*Oreochromis niloticus*) Fresh water	Tissue Body Viscera Environment	24	Culture**	20	*S.* spp.
Alexopoulos, A. et al. (2011)[99]	Greece	Aquaculture	Fish (species not described)		75	Culture**	1(1,43%)	*S.* spp.
Hudecova, K. et al. (2010)[100]	Hungary	Industry	Fish (*Cyprinus carpio* L.)	Tissue	360	Culture*	Nd	

Basti, A. et al. (2006)[13]	Iran	Aquaculture, market	Fish (*Alosa kessleri*, *Hypophthalmichthys molitrix*, *Liza aurata*)	Tissue	107	Culture*	1(0,93%)	Dublin
ALameer, A. et al. (2020)[101]	Iraq	Aquaculture, market	Fish (*Cyprinus carpio*)	Viscera	50	Culture**	20(40%)	*S.* spp.
Furushita, M. et al. (2003)[102]	Japan	Aquaculture	Fish (*Seriola lalandi*, *Thunnus thynnus*, *Seriola quinqueradiata*, *Seriola dumerili*)	Body<br>Viscera		Culture**<br>PCR	yes	Typhimurium
Terentjeva, M. et al. (2015)[103]	Latvia	Market	Fish (*Rutilus rutilus*, *Carassius carassius*, *Tinca tinca*, *Cyprinus carpio*, *Vimba vimba*, *Scardinius rythrophtalmus*) Fresh water	Tissue<br>Environment	235	Culture*	Nd	
Harakeh, S. et al. (2006)[104]	Lebanon	Market	Crab (species not described), Salt water, Fresh water, Sediment	Body<br>Environment	31	Culture*<br>PCR	15	Choleraesuis<br>*S.* spp.
Valenzuela-Armenta, J. et al. (2018)[105]	Mexico	Aquaculture	Fish (*Tilapia niloticus*) Fresh water	Tissue<br>Environment	126	Culture**	Nd	
Nesse, L. et al. (2005)[106]	Norway	Aquaculture	Fish (*Salmo salar L.*)	Body<br>Tissue<br>Viscera	295	Culture*	yes	Agona<br>Montevideo
Pyz-Lukasik, R. et al. (2018)[107]	Poland	Aquaculture	Fish (*Ctenopharyngodon*	Tissue	24	Culture*	Nd	

			idella, Aristichthys nobilis, Acipenser baerii, Silurus glanis)					
Antunes, P. et al. (2018)[22]	Portugal	Aquaculture, market	Fish (*Oncorhynchus mykiss*), Fresh water Sediment	Tissue Environment	53	Culture* PCR	14(26%)	Newport Linguere Guerin Abony
Al-Harbi, A. et al. (2006)[108]	Saudi Arabia	Aquaculture	Sediment	Environment		Culture**	4(3,81%)	*S*. spp.
Ntengwe, F. W. et al. (2008)[109]	Zambia	Aquaculture	Fresh water	Environment		Culture**	yes	*S*. spp.

Legend: *ISO 6579, FDA, AOAC or APHA, **Other descriptive methods; Nd = nondetected *Salmonella*.
